# The Multi-Pistil Phenomenon in Higher Plants

**DOI:** 10.3390/plants14071125

**Published:** 2025-04-04

**Authors:** Liang Chai, Cheng Cui, Benchuan Zheng, Ka Zhang, Yanling Li, Tongyun Zhang, Yongchun Zhou, Jun Jiang, Haojie Li, Jinfang Zhang, Liangcai Jiang

**Affiliations:** 1 Crop Research Institute, Sichuan Academy of Agricultural Sciences, Chengdu 610066, China; chailiang1982@126.com (L.C.);; 2 Environment-friendly Crop Germplasm Innovation and Genetic Improvement Key Laboratory of Sichuan Province, Chengdu 610066, China; 3 Key Laboratory of Tianfu Seed Industry Innovation (Co-Construction by Ministry and Province), Ministry of Agriculture and Rural Affairs, Chengdu 610066, China

**Keywords:** multi-pistil, MADS, candidate gene, yield, seed number, environmental factor

## Abstract

Correct floral morphology determines the accuracy of fruit formation, which is crucial for reproductive success in higher plants. Despite this, an abnormal, multi-pistil phenotype has been observed in the flowers of many plants. In this review, we gather information on the multi-pistil phenomenon in various species and highlight potential causes, as well as possible consequences, of the trait. Our assessment of the reported multi-pistil phenotype in rice (*Oryza sativa* L.), wheat (*Triticum aestivum* L.), tomato (*Solanum lycopersicum* L.), *Medicago*, sweet cherry (*Prunus avium* L.), rye (*Secale cereale* L.), and rapeseed (*Brassica napus* L. and *B. campestris* L.) leads us to conclude that hybridization and mutation are the main factors that give rise to this phenotype. We also delve into the inheritance patterns of the multi-pistil phenotype and factors that influence this trait, such as nuclear–cytoplasmic interactions, temperature conditions, and shading. Finally, we discuss the effects of multi-pistil flowers on the yield of these plants. This analysis increases our understanding of floral development and lays the foundation for the potential utilization of the multi-pistil trait to increase seed production in crops.

## 1. Introduction

Pistil development in plants is an intricate process controlled by a complex system of genetic and environmental factors [1]. The floral organs of dicotyledonous (dicot) plants typically have a four-whorled structure: sepals, petals, stamens, and carpels, from the outermost to the innermost whorl [2]. For example, in flowers of the model dicot plant Arabidopsis (*Arabidopsis thaliana*), the first whorl contains four sepals, the second contains four petals, the third contains six stamens, and the fourth contains one bicarpellate pistil. The flowers of monocotyledonous (monocot) plants are significantly different from those of dicot plants in terms of morphology and structures. For example, rice (*Oryza sativa* L.) florets consist of a lemma, a palea, two lodicules, six stamens, and a carpel.

However, abnormal flowers have been found in many species, among which multi-pistil flowers are quite striking. Flowers/florets with this trait develop more than the usual single pistil, which consequently develop into unexpected siliques/pods/grains/fruits. The trait has been discovered in many plants, such as rice [3,4,5,6], wheat (*Triticum aestivum* L.) [7,8,9,10,11,12], tomato (*Solanum lycopersicum* L.) [13], Medicago (*Medicago sativa* L. and *Medicago truncatula* Gaertn) [14,15,16], sweet cherry (*Prunus avium* L.) [17,18], rye (*Secale cereale* L.) [19], sorghum (*Sorghum bicolor* L.) [20], Japanese apricot (*Prunus mume*) [21], and rapeseed (*Brassica napus* L. and *B. campestris* L.) [22,23,24,25,26,27]. This phenotype has been described as multi-pistil [3] or two-floret spikelets [28] in rice, multiple grains in sorghum [20], and aggregate-siliquae [23] or multi-siliques [27] in rapeseed. Despite these different names, all of these plants produce abnormal flowers with increased numbers of pistils, which can lead to additional seeds and fruits. Since the number of seeds/fruits plays an important role in crop yield, this trait is worthy of investigation.

In this review, we summarize many reports focusing on the multi-pistil phenotype in plants including field crops and fruit trees. We describe the phenotypes and origins of multi-pistil mutants, analyze their inheritance patterns, discuss the factors influencing the development of this trait, and describe its effects on plant yield.

## 2. The Multi-Pistil Phenotype in Various Plants

Multi-silique pistil phenotypes have been reported more frequently in rice and wheat than in other crops, perhaps because these crops are important staple foods and have thus attracted more attention from researchers. Moreover, rice and wheat are annual plants that produce progeny via meiosis and pollination yearly. Since mutations and genetic recombination frequently occur during this process, rice and wheat are more likely to produce novel traits than perennial plants.

### 2.1. Rice

Rice is a staple food for more than half the world’s population, particularly in Asia, where it serves as a major source of calories and nutrition [29,30]. Due to its importance as a food crop worldwide, much research has focused on rice. A typical flower of wild-type rice, from the outer to inner whorls, contains a lemma and a palea, two lodicules, six stamens, and one pistil. However, several rice lines show different floral morphology and organ composition, including variable pistil numbers. Whereas a normal wild-type rice flower contains only six stamens and one pistil, the rice *mp3* mutant [3], derived from a cross between *indica* and *japonica* rice, produces two to four pistils in a floret, which generates two seeds in a spikelet at the mature stage.

Compared with the wild-type accession, *mp3* displays lower phenotypic values for filled grains per panicle, grain setting rate, and grain yield per plant, while it has increased 1000-grain weight. The *mp3* trait is controlled by a single recessive gene, which is located in a 30.6 kb region (between markers L3-135 and RM7576) on the short arm of chromosome 3.

Another mutant, also named *mp3* [6], was similarly identified among the progeny of a cross between *indica* and *japonica* rice and is also controlled by a single recessive gene. Based on the construction of an F_2_ population and the use of markers, the causative locus was mapped to the short arm of chromosome 11.

The *multi-floret spikelet 3* (*mfs3*) mutant [4], derived from ethyl methanesulfonate (EMS)-treated rice cultivar ‘XIDA 1B’, also displays more floral organs than is typical in some spikelets, with two lemmas, four marginal regions of palea, four lodicules, eight to ten stamens, and two pistils. In the *mfs3* mutant, the main body of the palea is severely degenerated. This trait is also controlled by a single recessive gene.

Zheng et al. [4] mapped the *MULTI-FLORET SPIKELET 3* (*MFS3*) gene using 426 F_2_ plants and identified the underlying gene, LOC_Os06g04540, which was mapped to chromosome 6 between markers RM19347 and RM19352. Further analysis revealed that the encoded protein downregulates *OsMADS1* and *FON1* and upregulates *OsIDS1* and *SNB*, which regulate spikelet development, and downregulates *REP*, which is involved in palea development. These findings, coupled with the discovery that LOC_Os06g04540 in the mutant lacks an 83 bp fragment and harbors a base substitution, suggest that LOC_Os06g04540 is the candidate gene for the *mfs3* trait.

In 1996, Nagasawa et al. described three rice mutants, *floral organ number 1* (*fonl*) and the alleles *fon2-1* and *fon2-2* [31], which showed increased numbers of floral organs, particularly stamens and pistils. The mutants contained an enlarged floral meristem, but the vegetative meristem was unaffected, suggesting that *FON1* and *FON2* function exclusively in the regulation of the floral meristem and not the vegetative meristem.

Another rice mutant named *abnormal floral organ number1* (*afon1*) [5] was discovered in an M2 population generated by EMS mutagenesis of *indica* cultivar ‘Zhenong 34’. The mutant trait of *afon1* is controlled by a single recessive gene. The *afon1* locus was mapped to the arm of chromosome 1, and a mutation causing an amino acid substitution was identified in the candidate gene LOC_Os01g67430.

The multi-pistil rice mutants *mp1* and *mp2* were also identified, and the underlying genes were mapped to chromosome 1 and chromosome 3, respectively [6,32].

### 2.2. Wheat

The “multi-ovary trait” was first reported in wheat in 1983 [33], with subsequent reports describing similar traits.

In 2003, Peng [12] described the common wheat line ‘Three Pistils’, which carries three pistils in a single floret. This trait is different from pistillody, in which other floral organs are transformed into pistil-like structures because all its pistils have the potential to develop into grains. Although the number of grains per spike is higher in the three-pistil mutant than in the wild type, single seed weight is lower [11,12]. Genetic analysis showed that the three-pistil trait is controlled by a single dominant gene. The candidate gene, *Pis1*, was mapped onto chromosome 2DL between markers *Xgwm539* and *Xgwm349* [11].

In order to fine-map the *Pis1* gene, Yang [8] constructed an F_2_ mapping population (CM28 × CM28TP) and used genotyping-by-sequencing single-nucleotide polymorphism (GBS-SNP) data and Kompetitive Allele-Specific PCR (KASP) assays. Two flanking SNP markers, M70 and M71, were identified that are tightly linked to the *Pis1* gene, with a physical distance of 3.40 Mb encompassing 127 protein-coding genes.

In subsequent studies, the molecular mechanisms behind this phenotype were investigated using the following lines: a three-pistil mutant (TP), a single-pistil TILLING mutant of TP (SP), the three-pistil near-isogenic line CM28TP, and the parental cultivar CM28. The authors determined that the deficiency of ARF5, encoded by a gene located in the *Pis1* region, might be responsible for the three-pistil phenotype in TP. ARF5 is a transcription factor that functions in floral organ initiation by directly activating critical regulators of floral development [34,35].

Guo et al. [9] described the wheat line DUOII, a multi-ovary line whose flowers contain two to three pistils and three stamens and can develop two to three seeds like normal wheat. This trait was found to be controlled by a dominant gene whose expression is determined by nuclear–cytoplasmic interactions, as described below. In a subsequent proteomic analysis of 2- to 6 mm young spikes of plants in F_1_ populations (DUOII × TZI and TZI × DUOII) [36], the researchers identified 90 differentially accumulated proteins (DAPs) involved in various functional pathways, such as chloroplast metabolism, nuclear and cell division. Among these proteins, Flowering Locus K Homology Domain and PEPPER play essential roles in the specification of pistil organ identity [36].

Li et al. [7] studied another multi-pistil wheat line named MA, which resulted from natural mutagenesis. The authors determined that the secondary pistils in this line are derived from extra stem cells that fail to terminate normally between the carpel primordium and the lodicule primordium. Based on the timing of pistil development in MA wheat, the researchers assigned these pistils as a main pistil and secondary pistils. The development of these pistils is not synchronous, and some secondary pistils cannot develop into grains after pollination. Some secondary pistils gradually shrink and adhere to the base of the grain formed by the main pistil following pollination. A BC_6_F_2_ population was produced from a cross between MA and Chinese winter wheat cultivar 77(2), which typically has one pistil. Comparative proteomic analysis using iTRAQ identified 334 DAPs.

Another example is *dms*, a dwarf, multi-pistil, male-sterile wheat mutant from ‘Zhoumai 18’, a popular wheat cultivar in Henan Province, China. The multi-pistil and male sterility phenotypes were found to be controlled by a single recessive gene locus [10].

### 2.3. Rye

In rye, Malyshev et al. observed multiple pistils in the *mp* mutant [19]. Unlike a normal floret, which consists of one pistil and three anthers, each flower in this mutant contains four pistils. The mutant locus was mapped to the centromere region of chromosome 7R.

### 2.4. Sweet Cherry

The multi-pistil phenomenon has also been reported in sweet cherry. Wang et al. [18] and Liu et al. [17] discovered that the MADS-box genes *PaMADS3/4/5/12* play important roles in regulating pistil development in sweet cherry. *PaMADS3/4/5* expression is sensitive to high temperatures, and *PaMADS3* is a heat-inducible transcription factor gene. Protein complexes formed by PaMADS3–PaMADS5 and PaMADS5–PaMADS4 contribute to multi-pistil formation at high temperature. A heat shock element was identified in the promoter of *PaMADS12*; this gene is expressed at much higher levels at 35 °C than at 25 °C in transgenic tobacco leaves. The authors proposed that high temperature during culture affects the expression of *PaMADS12*, triggering the development of multiple pistils per flower. In addition, Wang [37] found that heterologous overexpression of the *FRUITFULL* gene from sweet cherry (*PavFUL*) leads to multi-silique formation in transgenic Arabidopsis.

### 2.5. Medicago Plants

Alfalfa (*Medicago sativa* L.) is a significant forage crop for cattle, horses, goats, and other animals. Alfalfa flowers are arranged in short racemose inflorescences, and the corolla is papilionaceous, with five petals; a single flower contains 9 + 1 stamens and one pistil [14]. Here, “9 + 1 stamens” indicates that there are 10 stamens per flower: nine of these stamens form a single cannular structure outside the pericardium, and the other stamen is separate. Two natural multi-pistil mutants (M-fon1 and M-fon2) were identified in male-sterile alfalfa lines [15]. The stigma receptivity and ovule number of M-fon1 and M-fon2 are lower than those of normal plants. The pistils of M-fon1 also show a high degree of degradation. Zhang [14] identified the natural mutant *mip*, with two to three pistils in 45.8–70.3% of florets; the length of the pistils decreases with an increasing number of pistils.

In *Medicago truncatula* Gaertn, a bi-pistil mutant was identified among a transgenic population [16] harboring the alfalfa mosaic virus (AMV) coat protein (CP) gene in antisense orientation driven by the cauliflower mosaic virus 35S promoter. The bi-pistil transgenic plants contained two separate stigmas borne on two separate styles from a single carpel primordium. This is the first known report of such a mutation in *M. truncatula* [16]. The trait is controlled by a single recessive gene. The authors attributed this mutation to gene disruption caused by a T-DNA insertion rather than the altered function of the gene itself, since multiple copies of the T-DNA were observed in the mutant. Thus, the true causal gene whose function was interrupted, leading to the multi-pistil phenotype, is still elusive.

### 2.6. Sorghum

Sorghum, an important diploid grain crop, is widely used in food processing, brewing, and feed production [38]. Liu et al. [20] reported on a multiple-grain sorghum germplasm with unique spikelet structures, including multi-florets, multi-pistils, multi-stamens, and consequently double grains. Notably, this multiple-grain trait can be dominant or recessive depending on the genetic background. The authors used different multiple-grain materials as male parents: TX431 or Shuangli (Huaide) crossed with XihongzaoAB (normal flowers). The progeny of XihongzaoAB × TX431 showed a dominant multiple-grain trait, while in the progeny of XihongzaoAB × Shuangli (Huaide), this trait was recessive; based on the segregation of this trait in F_2_ populations from both crosses, this trait is controlled by a single gene. These findings suggest that the multiple-grain trait may be controlled by different genes.

### 2.7. Japanese Apricot

Japanese apricot (*Prunus mume* Sieb. et Zucc.) is a widely grown fruit tree in China. Shi et al. [21] found that *PmWUSCHEL* (*PmWUS*) is expressed at significantly higher levels in a multi-pistil cultivar than in a single-pistil cultivar during the pre-differentiation and differentiation stages.

### 2.8. Tomato

The multi-pistil phenomenon has also been reported in tomato [13]. Fruits of multi-pistil tomato resemble a few cherry tomatoes that have merged. When single-pistil plants are crossed with multi-pistil plants, the segregation of pistil types in the F_2_ population follows a double-recessive epistatic gene interaction, with a ratio of 9:7 (single: multi). In other words, if both loci are heterozygous or homozygous dominant, the plants will display only single-pistil flowers, which is different from other examples mentioned above.

### 2.9. Rapeseed

Finally, the multi-pistil trait has also been identified in the *Brassicaceae* family. This group of plants is characterized by several key features, including a flower consisting of four petals, six stamens, and one pistil. Important members of this family include rapeseed and Arabidopsis. Rapeseed comprises three types: *B. napus* (AACC), *B. juncea* (AABB), and *B. campestris* (AA). It is the second largest oilseed crop after soybean (*Glycine max*), providing 13.0–16.0% of global vegetable oil production [39]. Since the number of siliques per plant is an important parameter determining yield in rapeseed [40], it is worth exploring the multi-pistil phenomenon in this crop, since it may lead not only to an increased number of siliques per flower, but also an increased total number of seeds per plant.

Wu et al. [41] reported on the 90-12 mutant, with multiple siliques, identified among the progeny of interspecific crosses between *B. napus* and *B. campestris*. Flowers in the main inflorescence and upper branches contain two to three pistils, which develop into two or three siliques. The authors found that although plants with multiple siliques produce fewer seeds per silique, seed yields are higher than those from the control variety Zhongyou821. Further investigation revealed that the trait is controlled by three recessive genes [42].

Hu [24] observed multiple pistils in the *B. campestris* male-sterile line 29MA. In 29MA, the majority of flowers in each plant contain two to three pistils and four to five petals. Guan et al. [43] subjected *B. napus* seeds to ^12^C heavy ion beam irradiation and unexpectedly obtained mutant plants with multiple pistils per flower, which could then develop into double siliques; this mutation was considered to be dominant.

In the 1990s, Jiang et al. [22,23,44] studied various multi-silique rapeseeds derived from *B. napus* × *B. campestris* progeny produced at the Sichuan Academy of Agricultural Sciences. In these plants, each flower contains two to three independent pistils sharing the same receptacle, which then develop into two to three individual siliques [27] on one carpopodium. Thus, the seed yield of each carpopodium is much higher than that of normal plants. This trait is significantly affected by sowing time and environmental factors: the multi-silique trait totally disappears if plants are grown under different climates. Subsequently, one of these plants, named zws-ms, was studied in order to explore the underlying mechanism of the multi-silique trait. Chai et al. [27] constructed near-isogenic lines (NILs) and obtained two extreme DNA pools (a multi-silique pool and a single-silique pool). Using whole genome re-sequencing (WGR) based on bulked-segregant analysis (BSA), the authors identified two associated regions on chromosomes A09 and C08, respectively. In a subsequent series of studies [26,27,45], researchers analyzed genes with nonsynonymous mutations and frameshift mutations within these associated regions, as well as differentially expressed genes (DEGs), DAPs, and alternative splicing (AS) events between the two NILs. The authors identified various potential causal genes, including MADS-box genes known to be involved in flower development. Some of these genes show different expression levels between the two NILs, while others possess nonsynonymous SNPs or frameshift InDels. Further research on this topic is ongoing.

### 2.10. Arabidopsis

Due to its small genome, rapid lifecycle, and ease of cultivation, Arabidopsis is widely used as a model plant. Genes from other species have been introduced into Arabidopsis to investigate their functions in floral development. Bai et al. [46] introduced the *PmKNAT2/6-a* gene from Japanese apricot into Arabidopsis and found that its heterologous overexpression results in a multi-pistil phenotype. *PmKNAT2/6-a* is a class I *KNOTTED1-like homeobox* (*KNOX*) gene. AGAMOUS-like 24 (PmAGL24) directly binds to the promoter of *PmKNAT2/6-a* and regulates its expression. PmAGL24 is a member of the MADS-box family [46], and AG genes are known to be involved in the development of floral organs, especially carpels, ovules, and fruits [47].

Peng et al. [48] cloned *BnFUL* from *B. napus*, an ortholog of Arabidopsis *FRUITFUL* (*FUL*), and introduced it into Arabidopsis. Initially, the authors wanted to examine whether resistance to pod shattering would be enhanced in the transgenic lines, since they previously revealed [49] that this gene positively regulates this trait. Surprisingly, the heterologous expression of this gene not only enhanced resistance to pod shattering in Arabidopsis, but two of these lines also showed the multi-silique trait.

## 3. The Origin and Source of the Multi-Pistil Trait

The above descriptions suggest that several different factors can give rise to the multi-pistil trait, including natural mutations, induced mutations, transgenic expression, and distant hybridization.

The multi-pistil trait is observed in naturally occurring mutants, such as wheat MA lines [7] and alfalfa *mip* [14,50]. However, it should be noted that this type of mutation occurs completely randomly, and thus it is impossible for breeders to predict where and when they might identify this trait. As a result, natural mutation cannot be used as a reliable method for creating novel phenotypes in plants.

Induced mutations, including mutations produced by chemical mutagenesis or irradiation, can also generate a multi-pistil phenotype. EMS is the most frequently used reagent for producing mutations and can lead to one or several mutated sites. The mutations in the abovementioned *mfs3* [4] and *afon1* [5] rice lines are controlled by a single recessive gene located on chromosome 6 and chromosome 1, respectively. Irradiation can also result in plants with the multi-pistil trait, such as the multi-pistil rapeseed mutant produced by Guan et al. [43]. ^12^C heavy ion beam treatment of rapeseed seeds resulted in many mutations leading to various phenotypes, such as tumor-like roots, dwarf stems, light green spoon-shaped leaves, multi-pistil flowers, and yellow seeds. This indicates that radiation acts randomly on DNA, resulting in traits that cannot be predicted. However, inducing mutations is a more effective process for creating multi-pistil flowers compared with natural mutations.

By contrast, transgenic technology is a relatively well-targeted way of generating multi-pistil plants and identifying gene functions. For instance, Wang et al. [37] identified *PavFUL* as a candidate gene for the multi-pistil phenomenon in sweet cherry [17,18] and introduced this gene into Arabidopsis, obtaining multi-pistil plants.

Transgenic multi-pistil plants have also been obtained using genes whose functions have not been completely verified. For instance, when Peng et al. investigated the pod-shattering resistance conferred by *BnFUL* [49], they heterologously expressed this gene in Arabidopsis. In addition to obtaining three shattering-resistant transgenic lines as expected, the authors also obtained two multi-pistil lines, as described above [48]. Therefore, transgenic plants are another important resource for identifying multi-pistil plants, even though the functions of some genes used for transformation and how they regulate this trait are unknown. Nevertheless, bio-safety concerns are a current predicament. Since many countries are still cautious about genetically modified organisms (GMOs), transgenic crops are only grown in a limited number of countries.

Distant hybridization appears to be an important source of multi-pistil plants. Two multi-pistil rice mutants, which were coincidentally both named *mp3*, were identified by Liang et al. [3] in Chongqing and Jiang [6] in Hebei province, China. Both mutants were derived from crosses between *indica* and *japonica* rice. The former *mp3* mutant showed higher 1000-grain weight than wild-type rice [3]. In Sichuan, China, the *fon(t)* mutant [51] was discovered among the progeny of a cross between a diploid (Chunjiang 683) and a haploid (*SAR IV*-620-*A*) rice cultivar. From 1973 to 1982, researchers at the Sichuan Academy of Agricultural Sciences identified a total of 13 multi-pistil events in rapeseed. Except for the line named 37(3) of unknown origin, all the other lines were derived from interspecific crosses [22]. In addition, beginning in 1986, Wu et al. [52,53] intentionally crossed *B. napus* with *B. campestris*, obtaining a series of multi-silique materials. Therefore, both Wu et al. [53] and Jiang et al. [22] believed that interspecific crossing, especially *B. napus* × *B. campestris*, is an important source of multi-pistil rapeseed. This safe, traditional method used to create new germplasms has the potential to increase yields, although the underlying mechanism is complex and remains elusive.

## 4. Inheritance Patterns and Candidate Genes

In summarizing current information about multi-pistil mutants (Table 1), we noticed that most of these mutant phenotypes are controlled by recessive gene(s), except for the rice mutant *lf1* [54], wheat lines TP [11,12,55] and DUOII [9,36], and the radiation-induced rapeseed mutant [43]. The phenotypes of most of the recessive mutants are controlled by a single gene (Table 1); however, in rapeseed, this trait is controlled by two to three genes. Moreover, some situations are more complex: in sorghum, the multi-pistil traits can appear dominant or recessive, depending on the lines used for crossing, suggesting that different genes control this trait [20] depending on the material. In the tomato F_2_ population described by Mahfud [13], the segregation pattern follows that of a double-recessive epistatic gene interaction, with a ratio of 9:7 (single-pistil: multi-pistil).

In general, the candidate genes behind the multi-pistil trait have not been thoroughly studied, and there are few reports of the cloning and verification of these genes (Table 2).

**Table 1:** The sources and inheritance patterns of the multi-pistil phenotype in various plants.

Species	Accession Name	Source	Inheritance Pattern	References
Rice	*mp3*	*indica*–*japonica* cross	Recessive gene	[3]
	*mp3*	*indica*–*japonica* cross	Recessive gene	[6]
	*mfs3*	EMS chemical mutation	Recessive gene	[4]
	*afon1*	EMS chemical mutation	Recessive gene	[5]
	*fon(t)*	Diploid × haploid	Recessive gene	[51]
	TOR	*indica* C2 × Mianxiang 5B	Recessive gene	[56]
	*lf1*	Mutant	Dominant gene	[54]
Wheat	TP	Spontaneous mutant	Dominant gene	[11,12,55]
	DUOII	Common multi-ovary line	Dominant gene	[9,36]
	*dms*	Mutant from Zhoumai 18	Recessive gene	[10]
Rapeseed	Series of lines	*B. napus* × *B. campestris* or *B. napus* × *B. rapa*	Two recessive genes	[22,23,26,27]
	90-12	*B. napus* × *B. campestris*	Three recessive genes	[52]
	Not mentioned	Radiation mutation	Dominant gene(s)	[43]
*M. truncatula*	*bip*	T-DNA insertion	Recessive gene	[16]
Sorghum	TX431	Breeding	Dominant gene	[20]
	Shuangli (Huaide)	Breeding	Recessive gene	[20]
Tomato	-	Breeding	Double-recessive epistasis	[13]

One such underlying gene is *FLORAL ORGAN NUMBER1* (*FON1*) in rice. Mutations in the *fon1* locus cause a greater number of floral organs: in the *fon1-1* and *fon1-2* mutants, the number of pistils in a floret is substantially higher than that in the wild type, as is the stamen number. *FON1* encodes a leucine-rich repeat (LRR) receptor-like kinase sharing high similarity with CLAVATA1 (CLV1) in Arabidopsis [57]. The Arabidopsis *clv1* mutant contains more organs in all four whorls and can also have additional whorls [58].

*FON4*, also known as *FON2* [59], also regulates pistil number in rice flowers. Suzaki et al. [60] analyzed the function of *FON2* and found that the numbers of pistils in rice mutants *fon2-1*, *fon2-2,* and *fon2-3* were approximately 2.9-, 2.2-, and 2.0-fold greater, respectively, compared with the wild type. In the *fon2-1 fon1-1* and *fon2-1 fon1-2* double mutants, the flower phenotypes were very similar to that of the single mutant *fon2-1*, suggesting that *FON2* and *FON1* function in the same genetic pathway. Constitutive expression of *FON2* in transgenic rice results in sharply lower numbers of all floral organs. *FON2* encodes a small secreted protein containing a CLE domain, and according to its sequence, it is considered an ortholog of *CLV3* [60,61]. Chu et al. [61] analyzed *FON4* in the same year and reached the same conclusions. *FON4* and *FON2* were subsequently recognized as the same gene [59].

*LATERAL FLORET 1* (*LF1*) [54] influences the pistil number in rice, since the rice *lf1* mutant has three pistils per spikelet. *LF1* encodes a homeodomain leucine zipper class III (HD-ZIP III) transcriptional activator. HD-ZIP III genes form three strongly supported clades in angiosperms: *REV*, *PHB/PHV*, and *CNA/HB8* [62,63,64,65]. *LF1*, which belongs to the first clade, regulates embryo patterning, meristem function, vascular development, lateral organ polarity, and interfascicular fiber differentiation [54].

**Table 2:** Causal genes of the multi-pistil trait in various field crops and fruit trees.

Gene	Species	Characteristics	References
*FON1*	Rice	LRR receptor-like kinase; ortholog to *CLV1*	[57]
*FON4* (*FON2*)	Rice	CLE domain; ortholog to *CLV3*	[59,60,61]
*LF1*	Rice	HD-ZIP III	[54]
*WAG-2*	Wheat	MADS-domain	[66]
*PaMADS3/4/5/12*	Sweet cherry	MADS-box	[18,37]
*BnFUL*	Rapeseed	MADS-box, K-box	[48,49]

MADS-box genes also appear to be highly related to the multi-pistil flower trait. The MADS family is a large group of transcription factors containing a conserved MADS domain [67] that plays vital roles in regulating floral organ development [68]. Most members of the MADS-box gene family in plants contain four domains: the MADS domain, K-domain, I-region, and C-terminus. The MADS domain is highly conserved and is believed to be a DNA-binding site, while both the MADS and K domains likely participate in protein dimerization [12].

*PaMADS3/4/5/12* play important roles in regulating pistil development in sweet cherry [18]. Wang et al. [37] found that heterologously overexpressing *PavFUL* from sweet cherry, an ortholog of *FRUITFUL* and a member of the MADS family, leads to multiple silique formation in transgenic Arabidopsis, pointing to the ability of *PavFUL* to generate multi-pistil flowers.

As described above, the *BnFUL* gene was originally considered to enhance pod-shattering resistance in rapeseed [49], but when it was heterologously overexpressed in Arabidopsis, it produced multi-pistil flowers in some transgenic lines [48]. Although this result is unexpected and its molecular mechanism is unclear, it suggests that *BnFUL* is relevant to this phenotype. *BnFUL* is orthologous to *AGAMOUS* (*AG*) in Arabidopsis, which is also a MADS-box gene. Wei [66] determined that *WAG-2* regulates the multi-pistil trait in wheat. This gene is also an ortholog of *AG* from Arabidopsis.

By comparing the transcriptomes of *mp1* and wild-type alfalfa, Zhou et al. [69] determined that MADS-box genes (c68049_g1, c60978_g2) might participate in the development of multiple pistils. Other MADS-box genes, such as *OsMADS3* in rice [70,71], can also lead to the multi-pistil trait; OsMADS1 interacts with MFS3 [4], which regulates spikelet development.

## 5. Factors Influencing the Multi-Pistil Trait

Like other phenotypes, the multi-pistil trait is controlled by both internal and external factors, such as plant variety [17], cytoplasmic factors [9,36,72], temperature conditions [17,18,37], and shading [73].

In addition to the causal genes mentioned above, plant variety also influences multi-pistil formation. For example, in cherry, the rate of multi-pistil formation in ‘Tieton’ reaches 80–90%, which is much higher than that of ‘Lapins’ (20–30%) in Shanghai [17]. This may be attributed to some unknown genes from different genetic backgrounds.

The multi-ovary trait of DUOII is affected by a nuclear–cytoplasmic interaction [9,36]. To investigate this interaction, researchers conducted a reciprocal cross between DUOII and TeZhiI, which has the nucleus of common wheat and the cytoplasm of *Aegilops*, and constructed F_2_, F_3_, BC_1_, and BC_1_F_1_ populations. The heterogeneous cytoplasm of TeZhiI suppressed the expression of the heterozygous, but not homozygous, dominant gene conferring the multi-ovary trait. In addition to uncovering an interesting pathway regulating pistil formation in wheat, this finding sheds light on a nuclear–cytoplasmic interaction in this plant.

Among environmental factors, temperature plays a prominent role in the multi-pistil trait. Whiting and Martin [74] revealed that multi-pistil formation could be induced by high temperature during a key stage of flower bud differentiation in sweet cherry, resulting in polycarpy. Consistent with this finding, Wang et al. noticed that multiple carpels occur more frequently in the warmer climate of Shanghai than in the cooler climate of Dalian [18]. Moreover, heat stress leads to pistil hyperplasia in rice [75]. These results support our previous finding [22,23,26,27] that the multi-pistil trait is regulated by temperature. Specifically, the rapeseed line zws-ms exhibits stable multi-pistil formation in Chengdu, where it is relatively warm, but not in the colder region of Ma’erkang. These findings strongly suggest that higher temperature induces the formation of the multi-silique phenotype.

Shading can also affect the frequency of double pistils in sweet cherry: Beppu et al. [73] reported that the frequency of double pistils in flower buds and at anthesis was strongly decreased in ‘Satohnishiki’ sweet cherry with 78% artificial shading, likely due to the lower temperatures in the shade.

## 6. Impact on Yield and Agricultural Production

The ultimate aim of growing field crops and fruit trees is to achieve high yields, which are related to the number of seeds or fruits per plant. However, the impacts of multi-pistils on seed or fruit yield vary considerably among plants.

To date, there is no direct evidence that the multi-pistil trait can optimize the yield in wheat. However, as increasing the number of grains per spike has high potential for improving wheat yield [76,77], multi-pistil wheat is regarded as a resource for yield improvement.

Yield in rice is determined by three major components: panicle number per plant, grain weight, and grain/spikelet number per panicle [78]. Increasing the grain number per panicle is an important way of increasing yield in rice; thus, the three-floret spikelet has potential value for rice breeding [54].

Wu et al. [41,53] determined that the seed yields per plant in multi-silique rapeseed plants in two successive years were much higher than those of the control variety, and in two more successive years, the yields per hectare were also higher than the control. Jiang et al. [23] observed that multi-silique rapeseed plants possessed higher photosynthetic efficiency in their pericarps at the green pod stage compared with the wild type. Since photosynthesis in the green pericarp contributes significantly to seed yield, these findings point to the potential of this trait for rapeseed breeding [22,23].

The multi-pistil trait causes tomato fruits to function like cloves in garlic, allowing them to be split into smaller parts without damaging the whole fruit. This trait facilitates storage after the fruit has been partially consumed [13].

The multi-pistil trait appears to have a negative effect on fruit trees. In sweet cherry, polycarpy is considered to be a significant physiological disorder, decreasing the commercial value of the fruit [18]. Moreover, the formation of multi-pistil flowers in Japanese apricot reduces yield and quality [46].

In conclusion, we suggest that the multi-pistil trait tends to be beneficial in field crops, while it appears to be detrimental for fruit trees, as it decreases fruit yields and degrades fruit quality.

## 7. Conclusions

Multi-pistil flowers have been discovered and analyzed in various plant species. This trait has been most frequently studied in rice and wheat, perhaps because both these plants are annuals, so they reproduce seeds more often (annually) than perennial crops. Thus, genetic changes can occur more frequently in these crops. Moreover, rice and wheat represent important staple food crops, thus attracting a lot of attention. This phenotype is controlled by many factors, such as genes, nuclear–cytoplasmic interactions, and environmental factors. Researchers tend to consider this trait to be beneficial to field crops, such as rice, wheat, and rapeseed, while it is regarded as harmful to fruit yield. The effects of multi-pistil flowers on each type of plant require more analysis. The underlying molecular mechanisms are complex and still not clear. However, it is important to investigate the multi-pistil flower trait, as such studies could lay a theoretical foundation for studying floral development and may generate varieties with high yields.

## Data Availability

Not applicable.
